# Phase I clinical study of a novel lipophilic platinum complex (SM-11355) in patients with hepatocellular carcinoma refractory to cisplatin/lipiodol

**DOI:** 10.1038/sj.bjc.6601318

**Published:** 2003-10-28

**Authors:** S Fujiyama, J Shibata, S Maeda, M Tanaka, S Noumaru, K Sato, K Tomita

**Affiliations:** 1 Third Department of Internal Medicine, Kumamoto University School of Medicine, Japan

**Keywords:** hepatocellufalar carcinoma, SM-11355, lipiodol, trans-arterial chemoembolisation

## Abstract

SM-11355 is a platinum complex developed to treat hepatocellular carcinoma (HCC). It is administered via the hepatic artery, using a carrier, lipiodol, that consists of ethyl esters of iodized poppy seed oil. We have performed a phase I clinical trial of an SM-11355-lipiodol formulation in 11 HCC patients, in order to investigate the maximum allowable dose and to maximize the efficacy and safety of the drug in the treatment of HCC. The SM-11355 arterial infusion suspension was administered at doses of 6, 12 and 20 mg ml^−1^ in a maximum lipiodol volume of 6 ml. An antitumour efficacy rating of complete response was achieved for one patient and a partial response rating was achieved for a second patient, giving an overall response rate of 18.2%. Anorexia, nausea and vomiting, pyrexia, thrombocytopenia and increases in AST, ALT and total bilirubin were observed as adverse effects, but each was transient and each patient had recovered completely by 4 weeks after drug administration. Hence, we concluded that the maximum allowable dose was not reached in this study. Overall, our results suggest that SM-11355 is effective in treating HCC and we suggest that the dose for early phase II trials should be 20 mg ml^−1^.

It is widely known that lipiodol, which consists of ethyl esters of iodized poppy seed oil, selectively resides at tumour sites for a long period of time, following administration via the hepatic artery in chemoembolisation for hepatocellular carcinoma (HCC) (Nakakuma *et al*, 1983; Konno *et al*, 1984). In Japan, chemoembolisation for HCC is mainly used in advanced stage patients who cannot be treated with transcatheter arterial embolisation (TAE), or whose hepatic function standby capacity is low. Furthermore, Camma *et al* (2002) have reported that chemoembolisation has a significant therapeutic benefit, compared with more conservative therapies, based on the data from 18 radiotherapy centres. Several attempts have been made to take advantage of the properties of lipiodol, by coinfusion of lipiodol and anticancer agents such as zinostatin stimalamer (SMANCS®), doxorubicin, epirubicin, mitomycin C and cisplatin (Konno *et al*, 1983; Kanematsu *et al*, 1984; Ohishi *et al*, 1985; Yamashita *et al*, 1985; Kobayashi *et al*, 1986; Fukushima *et al*, 1988; Shibata *et al*, 1989). However, although this approach has played an important role in the treatment of HCC, the efficacy of formulations based on lipiodol extracts has not been optimized, partly because of incompatibilities of standard antitumour agents with lipiodol.

SM-11355, *cis*-[((1*R*,2*R*)-1,2-cyclohexanediamine-*N*,*N′*)bis(myr-istato)]platinum (II), is a platinum complex (Figure 1

**Figure 1:**
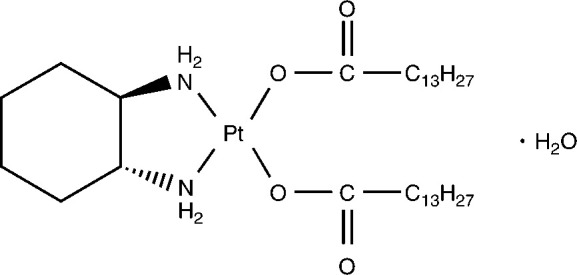
Molecular structure of SM-11355.

) and a novel therapeutic agent for the treatment of HCC. It is administered via the hepatic artery, using lipiodol as a carrier (Maeda *et al*, 1986; Kishimoto *et al*, 2000a, 2000b; Shimakura *et al*, 2002). SM-11355 is a derivative of cisplatin that acts as a prodrug, through the sustained release of the predicted active forms of the drug, cyclohexan-1,2-diamineplatinum (II) dichloride (DPC) and cyclohexan-1,2-diamineplatinum (II) dioxide (DPI). SM-11355 demonstrates excellent compatibility with lipiodol in forming a suspension and, when suspended in lipiodol, DPC and DPI are released into the aqueous phase, thus providing the antitumour effect (Maeda *et al*, 1986; Kishimoto *et al*, 1992). Here we report a phase I study that was conducted to determine the maximum allowable dose of SM-11355 and to examine the pharmacokinetics, efficacy and safety of the SM-11355 suspension in lipiodol.

## MATERIALS AND METHODS

### Patient selection

HCC patients were considered eligible for the study if they possessed the following characteristics: 20–75 years of age; at least one measurable tumour blush on angiography; no other therapeutic treatment was effective or suitable; an Eastern Cooperative Oncology Group (Oken *et al*, 1982) performance status of 0–2; adequate hepatic function (aspartate aminotransferase (AST) and alanine aminotransferase (ALT)⩽200 U l^−1^, total bilirubin⩽3.0 mg dl^−1^); adequate haematological function (white blood cells (WBCs)⩾3000 mm^−3^, platelets⩾50 000 mm^−3^, haemoglobin⩾10 g dl^−1^); and adequate renal function (serum creatinine⩽1.2 mg dl^−1^). All the HCC cases were recurrences with a history of cisplatin–lipiodol therapy. Patients who were not expected to survive at least 2 months were ineligible. Patients with active double cancers, who pronounced arteriovenous shunts or extensive extrahepatic metastasis, were also ineligible. Written informed consent was obtained from each patient and the study protocol conformed to the ethical guidance of the 1975 Declaration of Helsinki.

### Investigational agent

SM-11355 was obtained as a lyophilized product containing 20 mg of SM-11355 per vial, and it was stored in the dark under refrigeration below 5°C. Lipiodol, the SM-11355 carrier, contained 10 ml of ethyl esters of iodised poppy seed oil fatty acids per ampule, and was stored in the dark at room temperature. The SM-11355 arterial infusion suspension was prepared by suspending SM-11355 in lipiodol at the required concentrations.

### Method of administration

The SM-11355–lipiodol suspension was administered via intra-arterial infusion, using a catheter that was inserted into the nutrient vessels of the tumour under X-ray fluoroscopy. Administration continued until the suspension filled the tumour vessels, with a maximum dosing volume of 6 ml.

### Choice of dosage and maximum volume

When the SM-11355–lipiodol suspension was administered at a concentration of 6 mg ml^−1^ and a volume of 0.2 ml kg^−1^ as a hepatic arterial infusion to dogs, the toxic low dose was estimated to be 1.2 mg kg^−1^ or higher. In humans, this is equivalent to the administration of 10 ml of SM-11355–lipiodol suspension at a concentration of 6 mg ml^−1^. Based on these results, the starting concentration of SM-11355 in the phase I clinical study was chosen to be 6 mg ml^−1^. To determine an appropriate dosing volume, we considered the intra-arterial infusion of SMANCS®, a product similar to SM-11355. SMANCS® is administered suspended in SMANCS® infusion suspension carrier, a product equivalent to lipiodol, at a maximum volume of 6 ml. Lipiodol administration into the hepatic artery at a volume of 6 ml was confirmed to be safe prior to the study. The safety and efficacy of the SM-11355–lipiodol suspension mainly depends on the concentration, and the current study was designed to estimate a maximum allowable dose of SM-11355 through variation of the concentration. In doing so, we did not want to alter the volume, other than for reasons of tumour size, and a volume of 6 ml was chosen as the maximum volume from safety considerations. Up to this limit, the dose volume of the SM-11355 arterial infusion suspension was varied as necessary, based on tumour size.

Three dosage groups were used, at concentrations of 6, 12 and 20 mg ml^−1^, and at least three patients were enrolled in each group. The doses were chosen based on a modified Fibonacci method. The maximum allowable dose was defined as the minimum dose at which two out of three or more of the patients experienced adverse effects of grade ⩾2 in parenchymal organs (i.e., heart, liver, kidney, blood and lungs), according to the Japanese Society of Clinical Oncology Adverse Effects Form (Furue *et al*, 1986). Principally, SM-11355 was administered as a single intra-arterial infusion, but additional doses were permitted if the patient requested continued administration.

### Concomitant therapy

Concomitant treatment that might have affected the evaluation of the effects of SM-11355, such as the use of other anticancer drugs, radiation therapy, hormone therapy, etc., was not permitted. However, treatment for the purpose of alleviating symptoms resulting from adverse reactions occurring during the study was permitted at the discretion of the investigators.

### Measured parameters and time schedules

The background information of the patients was surveyed prior to the start of administration. Clinical laboratory tests were conducted prior to the administration and on days 1, 3, 7, 14, 21 and 28 after administration. In these tests, the following parameters were examined: general haematology (red blood cells (RBCs), haemoglobin, haematocrit, WBC, platelet count and differential WBC); serum biochemistry (total protein, albumin, total bilirubin, AST, ALT, lactic dehydrogenase (LDH), alkaline phosphatase (ALP), leucine aminopeptidase (LAP), *γ*-glutamyl transpeptidase (*γ*-GTP), total cholesterol, triglycerides, blood urea nitrogen (BUN), creatinine, electrolytes (Na^+^, K^+^, Cl^−^, Ca^2+^), amylase, P-amylase, lipase, C-reactive protein (CRP), blood glucose, prothrombin time (PT) and hepaplastin test (HPT)); urinalysis (protein, glucose, urobilinogen, creatinine and *N*-acetyl-*β*-D-glucosaminidase (NAG)); tumour markers (*α*-fetoprotein (AFP) and des-*γ*-carboxy prothrombin (DCP)) and electrocardiography. An evaluation of toxicities relating to adverse effects and clinical laboratory tests was made in accordance with the Adverse Effect Form of the Japanese Society of Clinical Oncology (Furue *et al*, 1986).

### Evaluation of antitumour effect

Antitumour response was evaluated from a computed tomography (CT) scan examined in the 2 weeks before administration and 1 week, 5 weeks and 3 months after administration. Evaluation was performed in accordance with the ‘Criteria for the Evaluation of Direct Efficacy in Treatment of Hepatocellular Carcinoma’ (Liver Cancer Study Group of Japan, 1994). Tumour size was measured as the sum of the products of the longest perpendicular diameters of all measurable lesions. Complete response (CR) was defined as disappearance or 100% necrosis of all tumours, partial response (PR) was defined as ⩾50% reduction and/or ⩾50% necrosis and minor responses (MR) was defined as 25 to 50% reduction and/or 25 to 50% necrosis. Progressive disease (PD) was defined as ⩾25% enlargement of the tumour. No change (NC) was considered as disease not qualifying for classification as CR, PR, MR, or PD. We regarded lipiodol accumulation in tumours as being an indication of necrosis. The effect of a drug on HCC is often evaluated by tumour necrosis, and the criteria referred to above include effects on tumour reduction and necrosis. In addition, Okusaka *et al* (2000) have reported that necrotic findings in CT have a correlation with necrosis determined histopathologically.

### Pharmacokinetics

The total platinum concentration in the plasma was measured in at least two patients in each dosage group before administration and at 1, 3, 5 and 24 h, 3 days, 1 and 3 weeks, and 3, 6, 9 and 12 months after administration.

### Discontinuation of treatment

Administration was discontinued if the patients or their legally authorised representatives requested discontinuation, or if the investigator deemed it necessary to discontinue administration due to serious adverse effects or progression of the disease.

## RESULTS

### Patient characteristics

The study was conducted from November 1994 to June 1995 at the Third Department of Internal Medicine, Kumamoto University Hospital. In all, 11 patients were originally enrolled into the study, but a grade 3 thrombocytopenia (grade 2 prior to administration) developed in one patient in the 20 mg ml^−1^ dosage group, and a further examination of the safety and efficacy was performed to complete the enrollment. Table 1

**Table 1:** Characteristics of patients

		**Number of patients**			
**Item**	**Value or description**	**Total**	**6 mg ml^−1^**	**12 mg ml^−1^**	**20 mg ml^−1^**
Evaluable for efficacy		11	3	3	5
Sex	Male/female	6/5	0/3	3/0	3/2
Age^a^	53–60	1	0	0	1
	61–70	7	3	3	1
	71–73	3	0	0	3
Tumour site^b^	PAMLC	1	1	0	0
	PAML	3	1	2	0
	PAM : AML	1 : 1	0	0	1 : 1
	AL : PL	2 : 1	0 : 1	0	2 : 0
	P	2	0	1	1
No. of tumours	Single	1	0	0	1
	2–3	5	1	1	3
	4–10	4	1	2	1
	10 or more	1	1	0	0
Tumour size (maximum diameter, mm)	⩽30	1	0	0	1
	31–40	4	0	1	3
	41–50	3	1	1	1
	51–60	1	0	1	0
	⩾61	2	2	0	0
Vascular infiltration	Vp_0_Vv_0_B_0_	3	0	0	3
	Vp_1_Vv_0_B_0_	8	3	3	2
Metastasis	No/yes	11/0	3/0	3/0	5/0
TNM stage	T_2_N_0_M_0_	1	0	0	1
	T_3_N_0_M_0_	1	0	1	0
	T_4_N_0_M_0_	9	3	2	4
Stage	II	1	0	0	1
	III	1	0	1	0
	IV-A	9	3	2	4
Cirrhosis	No/yes	1/10	0/3	0/3	1/4^c^
	Type B/type C/type B+type C	1/8/1	0/2/1	1/2/0	0/4/0
Clinical stage	I/II/III	5/5/1	0/3/0	2/1/0	3/1/1
Child classification	A/B/C	2/5/4	0/1/2	1/1/1	1/3/1
Oesophageal varices	No/yes/unknown	4/6/1	1/2/0	1/2/0	2/2/1
Previous history	No/yes	1/10	0/3	0/3	1/4
Complications	No/yes	0/11	0/3	0/3	0/5
Previous therapy^d^	No/yes	0/11	0/3	0/3	0/5
Initial/recurrence	Initial/recurrent	0/11	0/3	0/3	0/5
AFP (ng ml^−1^)	100 or more/less than 100	7/4	3/0	1/2	3/2
DCP (AU ml^−1^)	0.1 or more/less than 0.1	6/5	3/0	1/2	2/3

a The average age of all patients was 66.8 years and the average ages were 68.3, 65.3 and 66.8 years in the 6, 12 and 20 mg ml^−1^ groups.

b P=posterior segment; A=anterior segment; M=medial segment; L=lateral segment; C=caudal segment;

c chronic hepatitis;

d cisplatin–lipiodol therapy.

lists the demography of the patients. We note that all the patients had a history of cisplatin–lipiodol treatment and in seven of the 11 patients this treatment had been ineffective (Table 2

**Table 2:** Comparison of the effects of SM-11355 treatment and previous cisplatin/lipiodol treatment

**Patient no.**	**Effect of cisplatin/lipiodol**	**SM-11355 dose (mg ml^−1^)**	**Effect of SM-11355**
1	Ineffective	6	PD
2	Ineffective	6	PD
3	Effective	6	PD
4	Ineffective	12	PD
5	Ineffective	12	NC
6	Effective	12	CR
7	Ineffective	20	NC
8	Ineffective	20	PD
9	Effective	20	NC
10	Ineffective	20	PR
11	Unknown	20	NC

).

The 11 patients received SM-11355 at one of the following doses: three patients at 6 mg ml^−1^ (dose level 1), three patients at 12 mg ml^−1^ (dose level 2) and five patients at 20 mg ml^−1^ (dose level 3). All the patients were considered eligible for evaluation of toxicity and antitumour response. Complete response occurred in one patient (case No. 6) in the 12 mg ml^−1^ dosage group, and, at the request of this subject, a second dose was administered, in accordance with the protocol. At that point in time, a study was underway concerning the safety of the 20 mg ml^−1^ dose, and the concentration used in the second dose was 20 mg ml^−1^. The response of each patient is shown in Table 2, and compared with the efficacy of the previous cisplatin/lipiodol treatment.

### Toxicity

The major haematological toxicity was thrombocytopenia, which occurred in three of 11 patients, but no decreases in WBCs or haemoglobin were noted. At dose level 3, grade 3 (preadministration value: grade 2) and grade 2 (preadministration value: grade 1) thrombocytopenia were observed in two patients. In addition, there were four patients with a decrease in lymphocytes, four patients with an increase in eosinophils, one patient with an increase in basophils, one patient with a decrease in RBCs and one patient with a low haematocrit level (Table 3

**Table 3:** Haematological toxicities

		**Leucocytopenia (no. of cases)**					**Anaemia^b^ (no. of cases)**					**Thrombocytopenia (no. of cases)**				
		**Grade^c^**					**Grade^c^**					**Grade^c^**				
**Dose (mg ml^−1^)**	**No. of cases**	**1**	**2**	**3**	**4**	**Total (%)**	**1**	**2**	**3**	**4**	**Total (%)**	**1**	**2**	**3**	**4**	**Total (%)**
6	3	0	0	0	0	0	0	0	0	0	0	1	0	0	0	1
						(0)					(0)					(33.3)
12	3	0	0	0	0	0	0	0	0	0	0	0	0	0	0	0
						(0)					(0)					(0)
20	5	0	0	0	0	0	0	0	0	0	0	0	1	1	0	2
						(0)					(0)					(40.0)
Total	11	0	0	0	0	0	0	0	0	0	0	1	1	1	0	3
						(0)					(0)					(27.3)

a Other haematological adverse effect: four cases of decreased lymphocyte count, four cases of increased eosinophil count, one case of increased basophile count, one case of decreased RBC count, one case of decreased haematocrit. Case no. 6, second dose: decreased lymphocyte count, increased monocyte count.

b Anaemia: decreased haemoglobin.

c Severity of adverse effects based on Furue *et al* (1986).

). For patient No. 6, a decrease in lymphocytes and an increase in monocytes were noted following the second dose (see above).

Adverse effects observed with relatively high frequency included anorexia (six out of 11 patients, 55%), nausea and vomiting (seven out of 11, 64%) and pyrexia (11 out of 11, 100%). However, only one patient with anorexia and two patients with nausea and vomiting developed grade 3 symptoms. After the second dose, patient No. 6 experienced grade 2 pyrexia (Table 4

**Table 4:** Nonhaematological toxicities (signs and symptoms)

		**Anorexia (no. of cases)**				**Nausea/vomiting (no. of cases)**				**Pyrexia (no. of cases)**			
		**Grade^b^**				**Grade^b^**				**Grade^b^**			
**Dose (mg ml^−1^)**	**No. of cases**	**1**	**2**	**3**	**Total (%)**	**1**	**2**	**3**	**Total (%)**	**1**	**2**	**3**	**Total (%)**
6	3	1	1	0	2	0	2	0	2	0	3	0	3
					(66.7)				(66.7)				(100)
12	3	2	0	0	2	0	2	0	2	1	2	0	3
					(66.7)				(66.7)				(100)
20	5	0	1	1	2	0	1	2	3	0	5	0	5
					(40.0)				(60.0)				(100)
Total	11	3	2	1	6	0	5	2	7	1	10	0	11
					(54.5)				(63.6)				(100)

a Other nonhaematological adverse effects: one case of a cough (dose level: 6 mg ml^−1^). Case no. 6, second dose: pyrexia (grade 2).

b Severity of adverse effects based on Furue *et al* (1986).

). The nonhaematological laboratory test abnormalities consisted mainly of increases in total bilirubin, AST and ALT, which occurred in four patients (Table 5

**Table 5:** Nonhaematological toxicities (clinical laboratory results)

		**AST increase (no. of cases)**					**ALT increases (no. of cases)**					**Elevated t. bil. (no. of cases)^b^**				
		**Grade^c^**					**Grade^c^**					**Grade^c^**				
**Dose (mg ml^−1^)**	**No. of cases**	**1**	**2**	**3**	**4**	**Total (%)**	**1**	**2**	**3**	**4**	**Total (%)**	**1**	**2**	**3**	**4**	**Total (%)**
6	3	0	1	0	0	1	0	1	0	0	1	1	0	0	0	1
						(33.3)					(33.3)					(33.3)
12	3	0	0	0	0	0	0	0	0	0	0	1	0	0	0	1
						(0)					(0)					(33.3)
20	5	0	3	0	0	3	0	3	0	0	3	2	0	0	0	2
						(60.0)					(60.0)					(40.0)
Total	11	0	4	0	0	4	0	4	0	0	4	4	0	0	0	4
						(36.4)					(36.4)					(36.4)

a Other nonhaematological adverse effects: one case of decreased urine volume, one case of elevated *γ*-GTP, 10 cases of elevated CRP, four cases of elevated blood glucose, eight cases of elevated NAG in urine, two cases of a decreased hepaplastin test, two cases of elevated LDH, one case tested positive for glucose in urine, two cases of decreased serum potassium, one case of decreased prothrombin time, one case of elevated P-amylase, one case of elevated lipase. Case no. 6, second dose: elevated t. bil. (Grade 1), elevated CRP, elevated blood glucose, elevated NAG in urine, elevated serum potassium, elevated serum chloride, decreased serum calcium.

b t. bil.: total bilirubin increase.

c Severity of adverse effects based on Furue *et al* (1986).

). All patients had recovered completely from these effects by 4 weeks after administration.

### Response

Of the 11 patients, CR was observed in one patient, PR was observed in one patient, NC was observed in four patients and PD was observed in five patients. The overall response rate ((CR+PR/total number of subjects) × 100) was 18.2% (two out of 11). The response rates by dose levels are shown in Table 6

**Table 6:** Antitumour response

		**Response (no. of patients)**						
**SM-11355 dose (mg ml^−1^)**	**No. of evaluable patients**	**CR**	**PR**	**MR**	**NC**	**PD**	**CR rate (%)**	**CR+PR rate (%)**
6	3	0	0	0	0	3	0	0
12	3	1	0	0	1	1	33.3	33.3
20	5	0	1	0	3	1	0	20.0
Total	11	1	1	0	4	5	9.1	18.2

. For example, CR was observed in one of three patients at dose level 2 (33.3%), and PR was seen in one of five patients at dose level 3 (20%). For the CR patient at dose level 2, a second administration at 20 mg ml^−1^ was given, at the request of the patient and in accordance with the protocol.

### Pharmacokinetics

Figure 2

**Figure 2:**
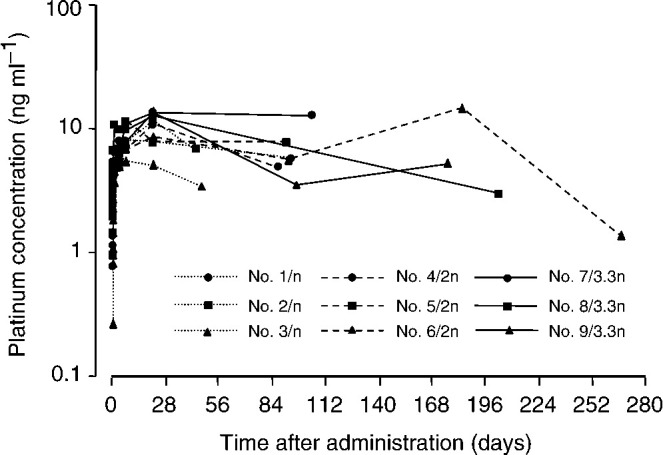
Change in plasma concentration of total platinum before and after SM-11355 injection. Measurements of plasma platinum concentration were taken before SM-11355 administration and at 1, 3, 5 and 24 h, 3 days, 1 and 3 weeks, and 3, 6, 9 and 12 months after administration. Three different doses were administered. Three patients (cases 1, 2 and 3) received SM-11355 at 6 mg ml^−1^ (dose level 1), three patients (cases 4, 5 and 6) received SM-11355 at 12 mg ml^−1^ (dose level 2) and five patients (cases 7, 8, 9, 10 and 11) received SM-11355 at 20 mg ml^−1^ (dose level 3).

shows the changes in platinum concentration in the plasma, measured in nine of the 11 patients. No clear relationship was found between the plasma platinum concentration and the total dose of SM-11355. The pharmacokinetic parameters were determined as follows: *C*_max_ 5.3–14.2 ng ml^−1^, *T*_max_ 7–183 days and *t*_1/2_ 18.4–707.2 days.

## DISCUSSION

In trans-arterial infusion therapy in HCC, doxorubicin hydrochloride, epirubicin hydrochloride, mitomycin C, cisplatin and SMANCS® have been used as anticancer agents using lipiodol as a carrier. It is particularly noteworthy that, with the exception of SMANCS®, all these agents are used as water-based preparations. It is difficult to prepare them as a suspension in lipiodol, which is an oil-based agent, and, at present, therapies involving lipiodol are clinically used by employing various homemade mixtures and suspensions. In contrast, SM-11355 was developed specifically to be used in a lipiodol suspension. This suspension has the unique characteristics of tumour selectivity and continuous release of active molecules, and it is expected to demonstrate better antitumour effects and improved safety, compared to formulations that are currently used.

We conducted a phase I trial for the purpose of estimating the maximum allowable dose in trans-arterial infusion of an SM-11355–lipiodol suspension, and to investigate the safety and efficacy in the treatment of HCC. Anorexia, nausea and vomiting, and pyrexia frequently emerged as adverse effects in SM-11355 administration at the lowest dose used. However, these are considered to be reactions that usually accompany hepatic intra-arterial infusion as a therapeutic modality, and the patients recovered within approximately 2 weeks following administration. The reactions were also judged to be less severe than those experienced with a cisplatin–lipiodol formulation (Shibata *et al*, 1989). Transient elevations of AST and ALT were also observed quite frequently, but all cases were complicated by chronic hepatitis or cirrhosis. Most of the patients had abnormal values prior to therapy, and these abnormalities may be characteristic of hepatic intra-arterial infusion. It is also possible that tumour necrosis may have played a role in the AST elevation. However, the treatment is given by direct drug injection into the hepatic artery and AST elevation was also found in patients who did not show necrosis. Therefore, we concluded that the drug had an effect on both tumour cells and normal hepatic cells, and hence we considered this effect to be an adverse reaction. Of those patients receiving a dose of 20 mg ml^−1^, one had grade 3 (preadministration value: grade 2) thrombocytopenia and one had grade 2 (preadministration value: grade 1) thrombocytopenia, based on the scale of Furue *et al* (1986). There were no other clinically significant adverse effects, and the adverse effects associated with SM-11355 were considered mild for an anticancer agent.

At a dose of 20 mg ml^−1^, adverse effects of grade 2 or higher were noted in three of five patients. With the exception of one patient with increased AST, all were one-grade fluctuations from preadministration grades, and we concluded that the maximum allowable dose had not been reached at a concentration of 20 mg ml^−1^. Since the maximum possible concentration that can be used for this preparation is 20 mg ml^−1^, we concluded that the recommended dose for use in early phase II clinical trials of SM-11355 should be 20 mg ml^−1^.

Plasma platinum concentrations were measured in nine of the 11 patients. No clear dose dependency was observed between the plasma platinum concentration and either the dosing concentration or total dose. The pharmacokinetic parameters of SM-11355 (*C*_max_ 5.3–14.2 ng ml^−1^, *T*_max_ 7–183 days and *t*_1/2_ 18.4–707.2 days) suggest that, in comparison with cisplatin–lipiodol (*C*_max_ 2–3 *μ*g ml^−1^, *t*_1/2_ 5–7 days) (Shibata *et al*, 1989), the plasma platinum concentration of SM-11355 is considerably lower (approximately 1/100–1/500 than that of cisplatin), but the half-life is much longer. We believe that this reflects the sustained release properties of the SM-11355–lipiodol formulation, in which SM-11355 migrates slowly from the lipiodol phase to the aqueous phase. In two patients receiving a dose of 20 mg ml^−1^, the lipiodol was washed out earlier than expected, and the retention rate at 3 months after administration was insufficient. However, tumour shrinkage was sustained for 3 months in these patients, although the tumour was not completely eliminated. Again, this probably reflects the ability of SM-11355 to exhibit sustained efficacy.

Finally, the antitumour response of one patient out of 11 was rated as CR at a dose of 12 mg ml^−1^ and one patient rated PR at a dose of 20 mg ml^−1^, for an overall response rate of 18.2%. In interpreting these statistics, it is important to note that patients treated at a low dose level were included, because this is a phase I trial. Furthermore, most of the patients had recurrent, advanced disease, with a history of largely unsuccessful cisplatin–lipiodol therapy. Hence, against this background, our results suggest that SM-11355 in conjunction with a lipiodol carrier shows excellent promise for the treatment of HCC. It is, however, recognised that this conclusion is based on data from a phase I study, and further studies will be needed to confirm the efficacy of SM-11355 for HCC.
